# Glucose-6-Phosphate Dehydrogenase, Redox Homeostasis and Embryogenesis

**DOI:** 10.3390/ijms23042017

**Published:** 2022-02-11

**Authors:** Po-Hsiang Chen, Wen-Ye Tjong, Hung-Chi Yang, Hui-Ya Liu, Arnold Stern, Daniel Tsun-Yee Chiu

**Affiliations:** 1 Graduate Institute of Health Industry Technology, College of Human Ecology, Chang Gung University of Science and Technology, Taoyuan 33303, Taiwan; bob79620@hotmail.com (P.-H.C.); claratania_louisregina@yahoo.com (W.-Y.T.); tychiu@mail.cgust.edu.tw (D.T.-Y.C.); 2 Research Center for Chinese Herbal Medicine, College of Human Ecology, Chang Gung University of Science and Technology, Taoyuan 33303, Taiwan; 3 Department of Medical Laboratory Science and Biotechnology, Yuanpei University of Medical Technology, Hsinchu 30015, Taiwan; 4 Department of Medical Biotechnology and Laboratory Sciences, College of Medicine, Chang Gung University, Taoyuan 33302, Taiwan; liuhy@mail.cgu.edu.tw; 5 Grossman School of Medicine, New York University, New York, NY 10016, USA; arnold.stern@nyulangone.org

**Keywords:** G6PD, G6PD deficiency, embryonic lethal, ROS, animal models

## Abstract

Normal embryogenesis requires complex regulation and precision, which depends on multiple mechanistic details. Defective embryogenesis can occur by various mechanisms. Maintaining redox homeostasis is of importance during embryogenesis. NADPH, as produced from the action of glucose-6-phosphate dehydrogenase (G6PD), has an important role in redox homeostasis, serving as a cofactor for glutathione reductase in the recycling of glutathione from oxidized glutathione and for NADPH oxidases and nitric oxide synthases in the generation of reactive oxygen (ROS) and nitrogen species (RNS). Oxidative stress differentially influences cell fate and embryogenesis. While low levels of stress (eustress) by ROS and RNS promote cell growth and differentiation, supra-physiological concentrations of ROS and RNS can lead to cell demise and embryonic lethality. G6PD-deficient cells and organisms have been used as models in embryogenesis for determining the role of redox signaling in regulating cell proliferation, differentiation and migration. Embryogenesis is also modulated by anti-oxidant enzymes, transcription factors, microRNAs, growth factors and signaling pathways, which are dependent on redox regulation. Crosstalk among transcription factors, microRNAs and redox signaling is essential for embryogenesis.

## 1. Introduction

The quality of an embryo and its growth warrant the existence and perpetuation of a species. Signaling events regulating cell proliferation, migration and differentiation with temporal and spatial precision are critical for healthy embryos. Defective embryos are subjected to destruction by various mechanisms [1]. Reduction-oxidation (Redox) status modulated by an imbalance of reactive species and anti-oxidants sensitizes signaling pathways, leading to altered cellular fates and embryonic defects [2]. Classic studies of embryogenesis have focused on the deleterious effects of oxidants on lipids, proteins and nucleic acids. Oxidants can serve as signaling molecules by initiating and promoting cell proliferation during embryonic development [3].

Glucose-6-phosphate dehydrogenase (G6PD) is the rate-limiting enzyme in the pentose phosphate pathway (PPP). G6PD is ubiquitously expressed in living organisms. The biochemical functions of G6PD include the oxidation of glucose-6-phosphate to 6-phosphogluconolactone and the production of the reduced form of nicotinamide adenine dinucleotide phosphate (NADPH). As a powerful biological reducing equivalent, NADPH supports reductive biosynthesis and maintains redox homeostasis. G6PD status is critical for life. Sufficient G6PD is necessary for normal cell growth and organismal survival. G6PD deficiency causes a wide range of pathophysiological effects, including growth arrest, senescence, cell death and embryonic defects [4]. 

NADPH can serve as a pro-oxidant for generating reactive oxygen species (ROS) via NADPH oxidase (NOX) and reactive nitrogen species (RNS) via nitric oxide synthase (NOS). Both ROS and RNS promote cell growth and differentiation [5]. G6PD is an important source of NADPH for the activity of NOX and NOS. The production of superoxide and nitric oxide is positively associated with G6PD status [6,7,8]. Because embryogenesis is coupled with metabolic alterations and energy consumption, ROS and RNS can be generated at supra-physiological concentrations. Anti-oxidant compounds and enzymatic systems can negate the harmful consequences of ROS and RNS. Among them, NADPH-dependent anti-oxidant enzymes, including thioredoxin reductase, aldo-keto reductase and glutathione reductase, are essential for reproduction and embryonic survival. 

Thioredoxin (Trx), a group of small redox proteins, reduces the formation of disulfide bonds in sulfhydryl residues. Oxidized Trx is then reduced by thioredoxin reductase (TrxR) using NADPH as an electron donor [9]. The importance of Trx in reproduction and fetal development is demonstrated by the embryonic lethality in Trx-knockout mice [10,11,12,13]. Carbonyl compounds, derived from oxidative modification of organic compounds, can react with thiols and amino acids leading to carbonyl stress [9]. The aldo-keto reductases catalyze the reduction of carbonyl groups to alcohols in an NADPH-dependent manner. This is associated with the protection of the male reproductive system [14]. 

NADPH is utilized by glutathione (GSH) reductase to regenerate the reduced form of GSH. Besides its role in anti-oxidative defense, GSH mediates various cellular processes by undergoing reversible oxidation with cysteine in protein targets, including kinases, phosphatases and transcription factors [15]. Glutathione peroxidase (GPx) utilizes GSH to detoxify peroxides. The expression of GPx4 in the testis and embryos suggests that it plays an essential role in male reproduction [16] and early embryonic development [13]. GSH is also linked with oxygen sensing through epigenetic regulation during development [17]. 

Oxygen has many roles in a biological system, including supporting metabolism and bioenergetics, maintaining redox homeostasis and supplying oxygen-based radicals as signaling molecules [18,19]. The evolution of metazoans is associated with increased levels of oxygen [20]. To adapt to the oxygen-rich environment, living organisms develop regulatory mechanisms to promote growth and survival, such as a cysteine-rich proteome. Approximately 3% of amino acids in proteins found in organisms are cysteines [21]. The complete development of the respiratory system is critical in an embryo and is, in part, redox sensitive [22]. The PPP functions as a key regulator for mediating hyperoxia-induced lung vascular dysgenesis at the late stages of embryogenesis. Hyperoxia-induced enhancement of the PPP causes abnormal proliferation of lung endothelial cells, dysmorphic vascular development and alveolar simplification in neonatal mice [23]. 

## 2. The Role of Redox in Humans and Other Species

Different animal models have been used in different redox studies because redox systems in all species are somewhat similar. In mammalian cells, the main source of intracellular ROS production are the NOX family (NOX 1-5, DUOX 1/2) [24] and the mitochondrial electron transport chain (ETC complexes I–IV, electron transporters ubiquinone and cytochrome c) [25,26,27]. Sequence comparisons and phylogenetic analysis indicate that *C. elegans* possesses twenty proteins closely related in sequence and length to TRX in *S. cerevisiae, H. sapiens*, and *D. melanogaster* [28]. Human mitochondrial TRXN2 (an ortholog with *C. elegans* TRX-2) is oxidized by mitochondrial-dependent superoxide, leading to the activation of the apoptosis signal-regulating kinase 1 (ASK-1) signaling pathway [29]. In humans, there are two thioredoxin genes, TXN and TXN2. In *S. cerevisiae*, thioredoxins are encoded by TRX1, TRX2 and TRX3 [30]. In addition, a family of anti-oxidant proteins, peroxiredoxins (Prx), are conserved in all types of organisms, including bacteria, plants, nematodes, and mammals. *C. elegans* peroxiredoxins-2 (CePrx2), a homolog of human PRDX2, is essential for normal growth and egg production [31]. 

In bacteria, fungi, worms and mammals, the disulfide reducing enzymes, GSH reductase and TrxR, are conserved. However, *D. melanogaster* does not have GSH reductase. It is replaced by TrxR, which results in the maintenance of a high GSH concentration [32]. Glutaredoxins (GLRX) are redox enzymes that use glutathione as a cofactor for reducing protein disulfides. The mammalian GLRX3/PICOT (ortholog of *C. elegans* GLRX-3) is involved in immune cell activation, development of cardiac hypertrophy, embryonic development and post-embryonic growth. GLRX3 also functions as an iron-sulfur protein [28]. Another redox compound, protein disulfide isomerase (PDI), which is found in the endoplasmic reticulum (ER), catalyzes the formation (oxidation), breakage (reduction) and rearrangement (isomerization) of disulfide bonds between cysteine residues within proteins. PDI-1, PDI-2 and PDI-3 are important for embryogenesis, viability and formation of the extracellular matrix in *C. elegans* [33].

## 3. G6PD Studies in Embryonic Stem Cells and Animal Models

**Figure 1:**
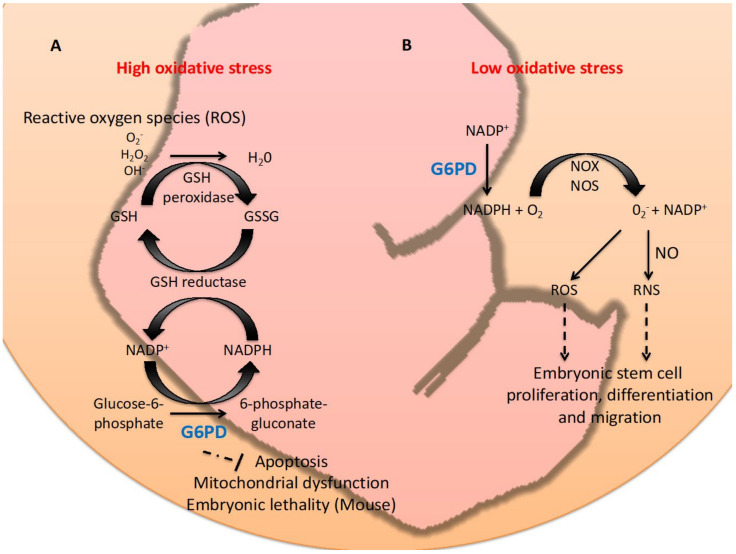
Graphic summary of the dual role, anti-oxidant or pro-oxidant, of G6PD in embryogenesis. Panel **A**: Under high oxidative stress conditions (supra-physiological), G6PD acts as a key anti-oxidative enzyme by eliminating ROS (O_2_^-^, H_2_O_2_ or OH^-^) via production of NADPH and regeneration of GSH. This mechanism prevents embryonic cells in animal models from undergoing apoptosis, mitochondria dysfunction and embryonic lethality in animal models. Panel **B**: G6PD can act as a pro-oxidant in low oxidative stress conditions (eustress) by providing NADPH for NOX/NOS in producing ROS or RNS, which are required for ESCs proliferation, differentiation and migration.

G6PD has a pro-survival role in cells and organisms [5]. How an enzyme involved in carbohydrate metabolism and redox homeostasis regulates cell survival and reproduction is largely unknown due to the biological complexity (Figure 1). 

### 3.1. Embryonic Stem Cells

Upon targeted inactivation of G6PD by homologous recombination, male mice embryonic stem (ES) cells are viable; however, they are very sensitive to oxidative stress [34]. Despite normal proliferation in vitro, G6PD-depleted ES cells are prone to apoptosis and mitochondrial dysfunction when challenged with oxidants. This can be ascribed to intracellular GSH levels [35]. G6PD-deficient ES cells can differentiate into many cell types, while G6PD-deficient erythroid cells undergo apoptosis upon hemoglobin switching [36]. This is caused by elevated oxidative stress due to enhanced release of oxygen from hemoglobin. Despite delaying apoptosis by the reducing agent, NAC, or a pan-caspase inhibitor zVAD-fmk, complementation of the G6PD gene into G6PD-deficient ES cells prevents cellular demise [36]. 

Cre-lox recombination-derived G6PD-deficient mouse ES cells are sensitive to oxidative stress [37]. Diamide, a potent thiol scavenger, depletes NADPH and GSH by oxidation and decreases survival in G6PD-deficient ES cells. While wild type ES cells rapidly restore the NADPH/NADP^+^ ratio and PPP activity upon diamide challenge, G6PD-deficient ES cells fail to do so. This indicates that the activation of G6PD is the major NADPH source in response to oxidative stress. 

### 3.2. Mouse

The importance of G6PD in embryogenesis has been shown in mouse studies [38]. G6PD(-) ES cells are used to inject into mouse blastocysts to produce chimeric mice. The first generation (F1) G6PD(+/-) heterozygotes obtained from the cross between normal females and chimeric males are healthy and fertile. Only normal G6PD males can survive when F1 G6PD(+/-) females are bred with normal males. Hemizygous G6PD(-) male embryos fail to grow at E.7.5, a critical stage for the development of the blood circulation. These embryos eventually die at E10.5. Likewise, heterozygous G6PD(+/-) females display tissue abnormalities and increased apoptotic cells. At later stages, necrotic cells appear and the embryos die between E11.5 and E12.5. Both G6PD(-) and F2 G6PD(+/-) embryos show apoptotic cells in the allantois, indicating a disturbance of placenta differentiation. The lethality of G6PD-deficient mouse embryos is ascribed to oxidative damage after the establishment of the blood circulation, thereby affecting placental function. Hence, G6PD plays a protective role against oxidative stress in the placenta to support the growth of trophoblast during embryogenesis. A comparative study shows that female mouse embryos contain less H_2_O_2_ and survive better than male embryos under heat stress [39]. The slow-growing female embryos display a higher level of G6PD transcripts. Upon G6PD inhibition, the differences caused by ROS between male and female embryos are absent, suggesting a protective role of G6PD in differential sensitivity of heat-induced oxidative stress during embryogenesis. 

### 3.3. Zebrafish

A transparent embryo and ex utero development make zebrafish an ideal model organism for studying embryogenesis. A G6PD morpholino knockdown zebrafish has been established due to the lack of significant hemolysis in G6PD-deficient mice upon oxidant challenge [40]. G6PD morpholino reduces G6PD activity in zebrafish leading to rapid hemolysis and pericardial edema. Titration of G6PD morpholino in zebrafish embryos does not cause edema and hemolysis until challenged with pro-oxidant compounds, including menthol, 1-naphthol and primaquine [40]. In addition to the edema and reduced hemoglobin in G6PD morphants, 1-naphthol increases ROS generation and erythrocyte apoptosis. Zebrafish embryonic defects caused by G6PD deficiency, including the decreased epiboly rate and elevated cell shedding at the embryo surface, are mediated by the epithelial-mesenchymal transition (EMT) pathway [41]. E-cadherin, encoded by *CDH-1*, regulates cell movement and tissue formation during early embryogenesis in zebrafish [42]. Reduction of E-cadherin decreases cell adhesion and the epiboly rate. Complementation of human *G6PD* or *CDH-1* cRNA reduces embryonic defects and the expression of epithelial surface markers, E-cadherin and β-catenin, in G6PD-deficient zebrafish embryos [41]. G6PD deficiency in zebrafish impairs redox signaling mediated by NADPH oxidase-4 (NOX4) and down-regulates Smad3 and miR-200b, which are involved in embryonic stem cell differentiation and stability [43,44]. These findings indicate that G6PD plays a cytoregulatory role in appropriating redox homeostasis for modulating embryogenesis.

### 3.4. Nematode

In order to decipher the common mechanism underlying early embryogenesis, a simple invertebrate animal model may be suitable for large-scale analyses using cell biology, molecular biology and genetic approaches. A pilot research program led by Sydney Brenner sought an organism with the potential to identify each gene involved in development and to trace the lineage of every cell. *Caenorhabditis elegans*, a free-living soil nematode, has been chosen because of several advantages [45]. *C. elegans* has become a favorite model of embryologists because its embryo and cuticle are transparent, thereby benefiting microscopic examination. *C. elegans*, approximately 1 mm in size, has a short duration of embryogenesis (16 h) and lifecycle (3 days) as well as a life span (3–4 weeks), which is ideal for investigation of the gene regulatory network in embryogenesis, development and aging.

The high degree of conservation of G6PD between the nematode and vertebrate counterparts indicates that G6PD in *C. elegans* is a functional homologue of vertebrate G6PD. A G6PD-deficient *C. elegans* established by RNA interference knockdown displays enhanced apoptotic germ cells and less embryo production, indicating impairment of oogenesis [46]. Similar to mouse and zebrafish models, G6PD-deficient *C. elegans* shows a dramatic reduction of hatched embryos. Such embryonic defects are possibly due to increased ROS and DNA oxidative damage, leading to altered MAPK signaling [46]. 

Further characterization of G6PD-deficient *C. elegans* embryos reveals that their membrane function is impaired. These membrane abnormalities in *C. elegans* embryos, including enhanced permeability to fluorescent dyes and loss of barrier structural integrity, as well as abnormal polarity and cytokinesis, are associated with altered lipid metabolism [47]. A significant increase of lysoglycerophospholipids is associated with the activation of calcium-independent phospholipase A2 (iPLA), possibly mediated by the imbalanced redox status, including decreased NADP levels and increased lipid peroxidation. Suppression of multiple iPLA alleviates defective permeability and polarity. 

Transient treatment of tert-butyl hydroperoxide (tBHP), an inducer of lipid peroxidation decreases brood size and increases germ cell apoptosis [48]. tBHP stimulates malondialdehyde (MDA) generation and up-regulates iPLA activity, thereby interfering with oogenesis and embryogenesis. These findings indicate that G6PD deficiency-derived lipid peroxidation enhances germ cell demise, leading to embryonic lethality, and demonstrates that G6PD is required for maintaining membrane stability and lipid homeostasis through redox balance during early embryonic stages.

## 4. The Redox Role of G6PD in Cellular Migration and Differentiation during Embryogenesis

Oxidative stress differentially influences cell fate and embryogenesis. Physiological levels (eustress) of ROS generated during embryogenesis promote proliferation and differentiation of cells and are required for normal development in the blastocyst stage and neuronal differentiation of embryogenesis [49]. ROS is involved in crucial processes, such as embryonic blood stem cell development [50,51] and differentiation of embryonic cardiomyocytes [52,53,54]. ROS can affect stem cell fate and generate stem cells, as seen with the differentiation of mesenchymal stem cells (MSC) to adipocytes caused by the enhancement of intracellular ROS through NOX4-mediated H_2_O_2_ signaling [55]. Increased ROS beyond physiological levels (oxidative stress) causes apoptosis, whereas even higher ROS levels lead to necrosis [56]. This suggests that redox-dependent signaling plays a critical role in cell migration and cell differentiation during embryogenesis (Figure 1).

### 4.1. Involvement of G6PD in Cell Migration 

Cell migration occurs during embryogenesis. In gastrulation, groups of cells inside blastocysts migrate collectively to form the three layers of the embryo (ectoderm, mesoderm and endoderm) that eventually migrate to target locations, where they differentiate and form various tissues and organs [57]. The collective cell migration requires coordination and teamwork between migrating cells, which interact with the extracellular matrix of other tissues and respond to chemotactic signals generated in the surrounding environment [58]. 

One of the signals that influence cell migration during embryogenesis is redox signaling, which can alter cell fate, leading to structural and functional changes in the developmental processes. Disruption of redox signaling during organogenesis induces adverse outcomes, such as defective cell migration, premature cell differentiation, apoptosis, alterations in cell proliferation and cellular polarity [2]. 

Redox signaling influences cell migration during embryogenesis through different mechanisms in different animals. Up-regulation of ROS induced by ethanol decreases hemangioblast migration from the posterior primitive streak to the area of opaca in the early chick embryo via the FGF/VEGF/PDGF signaling pathway [59]. In zebrafish, glutaredoxin 2, the vertebrate oxidoreductase protein modulating the intracellular thiol pool, is required for heart development. Knockdown of glutaredoxin 2 disturbs migration of the cardiac neural crest cells, leading to apoptosis [60]. Upon knockdown of superoxide dismutase 1, elevated ROS impairs embryonic primordial germ cell migration and adhesion in cellular blastoderm embryos of *Drosophila melanogaster* [61]. 

### 4.2. Contribution of G6PD to Cell Differentiation

Cell differentiation is closely associated with cell proliferation in a developing embryo. Embryonic differentiation occurs after zygote cleavage. The pluripotent ES cells differentiate into many cell types followed by organizing into tissues and organs [62,63]. Cell differentiation in the developmental process is influenced by redox signaling. ROS generated by osteoclasts is important for differentiation and the resorption of bone tissue [64]. Undifferentiated pre-adipocytes have a high level of GSH, whereas the GSH level drops and GSSG increases upon differentiation [65]. 

NOX4-dependent ROS production regulates cardiac differentiation through the p38 MAPK signaling pathway [66]. Increased ROS by NOX4 is involved in adipocyte differentiation via CREB (cAMP response element-binding protein) in mesenchymal stem cells [55]. Enhanced ROS production occurs during carbon monoxide-induced neuronal differentiation in SH-SY5Y cells. This event stimulates the PPP to increase NADPH production and affect cell differentiation [49]. 

## 5. Embryogenesis Regulated by Other Redox-Related Genes, Transcription Factors, MicroRNAs, Growth Factors and Signaling Pathways

**Figure 2:**
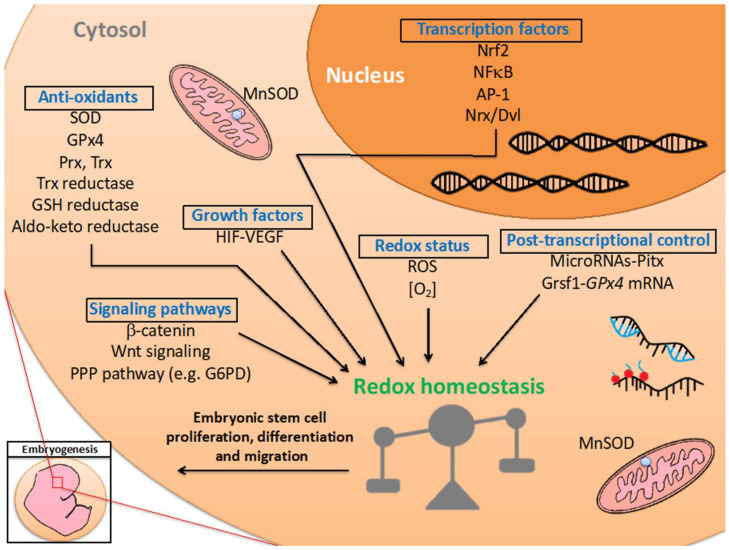
Regulation of redox homeostasis in embryogenesis. Redox-related factors, including anti-oxidant enzymes, transcription factors, post-transcriptional control, growth factors, signaling pathways and redox status, support embryogenesis and the developmental timeline through maintaining redox homeostasis. The biological function of these factors are discussed in the main text and summarized in Table 1.

Embryogenesis is a precisely controlled biological process for reprogramming the metabolic state of the cell and driving a single cell to a mature organism [67,68]. Embryogenesis is closely associated with activation and/or repression of genes within a developmental timeline [69,70]. This process is tightly and precisely regulated by various factors, such as transcription factors, microRNAs, growth factors and signaling pathways [71,72,73,74,75] (Table 1) (Figure 2). Redox reactions are essential for providing energy production and for regulating oxidative stress. Sufficient energy production and an appropriate redox environment are required for establishing a proper milieu for the embryo. Metabolically generated oxidants and associated anti-oxidant defenses participate in the initiation of certain outcomes during embryogenesis [76].

### 5.1. Anti-Oxidant Enzymes

Several anti-oxidant enzymes are involved in embryogenesis, such as superoxide dismutase (SOD), GPx, Prx and catalase (CAT). These anti-oxidant enzymes catalyze the decomposition of hydrogen peroxide into water and oxygen [28].

SOD controls ROS levels by converting superoxide to hydrogen peroxide, which can be detoxified to water by catalase and glutathione peroxidase [106]. Protein and mRNA of SOD are expressed during bovine embryogenesis [107]. The good quality of oocytes and embryos exhibits high SOD activity [108]. SOD also prevents the motility loss in mouse sperm and protects embryos from oxidative stress [109]. Newborn mice with sod-2^-/-^ deficiency show neonatal lethality after 4–5 days. Lacking SOD activity can be fatal in newborns [73]. These findings reveal the importance of ROS levels being regulated by an antioxidative enzyme in embryogenesis. 

GPx reduces H_2_O_2_ to H_2_O using GSH as the electron donor. One of the GPx isozymes, GPx4/PHGPx (phospholipid hydroperoxide glutathione peroxidase), reduces lipid hydroperoxide in cell membranes and is involved in gastrulation. GPx4 expression is up-regulated by G-rich RNA Sequence Binding Factor 1 (Grsf1) through a post-transcription mechanism. Grsf1 recruits GPX4 mRNA to translationally activate polysome fractions by targeting the 5′ untranslated region (UTR) of GPx4 mRNAs. Overexpression of GPX4 mRNA rescues Grsf1 deficiency-induced developmental retardation of embryos. This post-transcriptional regulation links the anti-oxidant enzyme, GPx4, to embryogenesis [102]. Homozygous GPx4-deficient mice die in utero at midgestation between 7.5 and 8.5 days post coitum because of retardation in brain development and abnormal heart formation [13,80]. However, the heterozygotes GPx4-deficient mice are viable, fertile and appear normal, in spite of having decreased levels of cytosolic, mitochondrial and nuclear GPx4 mRNA and proteins. These heterozygote GPx4-deficient mice are more susceptible to various oxidative stressors [13]. Another GPX isozyme, GPx3, is required for posterior embryogenesis in *Xenopus laevis*. GPx3 knockdown enhances cell death and decreased cell proliferation in the tail region through the Wnt/Notch/FGF signaling pathway [110]. 

Within the embryo, the nervous system is not completely developed [111]. Redox signaling contributes to the development of the nervous system, such as axonal growth and neuronal differentiation [112]. Redox imbalance can negatively affect embryonic neural development. Silencing GPx4 expression by RNA interference leads to a hindbrain developmental defect in mice (rhombomeres 5 and 6 impairment) [80]. SOD2 deficiency results in increased ROS levels, abnormal brain morphology and axonal aberration in *Drosophila* [79]. This suggests that embryonic development of the nervous system is redox sensitive.

Prx removes ROS through conserved reactive cysteines residues by which H_2_O_2_ is reduced. The mammalian Prxs affect gamete maturation, fertilization and embryogenesis. The deficiency of Prx does not have lethal consequences for embryogenesis. Among the six members of Prxs, Prx2, Prx3 and Prx5 influence embryogenesis [81]. Prx2 stimulates the development of the blastocyst by reducing intracellular ROS and increasing embryonic mitochondrial activity [82]. Prx3 is one of the candidate genes that predict embryo quality. Lower expression of Prx3 is associated with high-quality embryos [83]. *Drosophila* peroxiredoxin 5 (dPrx5) is essential in the early stages of embryogenesis [84]. *C. elegans* with Prx-2 deficiency have a reduced brood size and retarded development. The CePrx2 only affects two types of pharyngeal neurons, which are single pharyngeal interneuron 14 and the sensory interneuron 12 [31].

Catalase is found in all living organisms. One catalase molecule can reduce millions of H_2_O_2_ molecules to yield H_2_O and O_2_. The peroxisomal CTL-2-deficient *C. elegans* has a lower egg-laying capacity and shorter life span [113]. Embryonic catalase protects the embryo from excessive ROS production. The lack of catalase promotes structural embryopathies in C57BL/6 mice [114,115,116,117].

### 5.2. Transcription Factors

As key regulators of various cellular processes, transcription factors activate downstream target genes by enhancing transcription activity [118]. Transcription factors can sense cellular redox status, including NF-E2-related factor 2 (Nrf2), Nuclear factor kappa-light-chain-enhancer of activated B cells (NF-κB), Activator Protein-1 (AP-1) and hypoxia-inducible factor (HIF), thereby contributing to the regulation of cell proliferation, differentiation and apoptosis in embryos [76].

Nrf2 regulates the adaptive response to oxidative stress [119]. The evolution of Nrf2 is correlated with increasing atmospheric oxygen [120]. Nrf2 binds with genes containing the anti-oxidant response element (ARE) [121]. Nrf2 also serves as a master regulator for self-renewal, pluripotency and differentiation in ES cells [122]. In mesodermal development, multipotent stromal cells (MSCs) differentiate to osteoblasts, chondrocytes, myocytes and adipocytes [123]. Overexpression of Nrf2 in MSCs enhances oxidative stress resistance, cell survival and osteoblastic differentiation [87,88]. In early embryogenesis in mice, the Nrf2-associated cell cycle transition from the G2 to the M phase is inhibited by brusatol (a quassinoid isolated from *Bruceajavanica*), and this inhibition is dependent on the cyclin B-CDK1 (cyclin-dependent kinase I) complex [89]. This indicates that a dual role exists for Nrf2 in maintaining redox homeostasis and modulating embryogenesis. 

NF-κB regulates gene expressions, including that related to pro-inflammatory cytokines (IL1, IL2, IL6, IL8 and TNFα), adhesion molecules (VCAM-1, ICAM and E- and P-selectin), apoptotic regulators (Bcl-XL and c-IAPs), growth factors (G-CSF) and redox-related enzymes (COX2, iNOS, SOD, 12-LOX and GSH-synthase) [76]. Activation of NF-κB in response to stress, such as hypoxia or oxidative and nitrosative stress, results in the modulation of downstream gene expression [124]. During embryogenesis, loss-of-function of NF-κB leads to embryonic lethality at E15 and apoptosis of liver parenchymal cells in mice [90]. 

AP-1 regulates various gene expressions and controls proliferation, differentiation and apoptosis in response to cellular stress [125]. AP-1 is activated by the upstream MAP-kinase (p38, ERK and JNK) signaling pathways [126]. While NF-κB modulates the postnatal stage, AP-1 regulates embryogenesis in the prenatal stage. Knockout of AP-1 results in embryogenic defects in mice, including embryonic lethality, abnormal liver erythropoiesis, hepatogenesis and the development of the placenta and yolk sac, chondrocytes and extra-embryonic tissues [91,92,93,94,95].

### 5.3. Redox-Related Post-Transcriptional Controls

Post-transcriptional regulation plays a crucial role in redox-dependent developmental processes. Myogenesis is an important biological process for forming skeletal muscular tissue during embryogenesis [127]. Loss of function of two transcription factors, *Paired*-like homeodomain transcription factor 2 gene (*Pitx2*) and *Pitx3*, leads to abnormal accumulation of ROS in mouse myogenesis [100]. The Pitx-MicroRNA (mir-15b, mir-23b, mir-106b and mir-503) pathway regulates myoblast proliferation, demonstrating that the crosstalk of transcription factors, microRNAs and redox signaling is essential for embryogenesis [101]. 

The basic helix loop helix (bHLH) and Per-ARNT-SIM (PAS) transcription factor superfamily proteins of HIF are essential in developmental programs, such as trophoblast proliferation and differentiation, morphogenesis of the developing heart, chondrogenesis and myocardium development [96,97,98,99]. They activate various downstream target genes via the hypoxia response element in the enhancer region. Among three HIF-α subunits, HIF-1α is ubiquitously localized in developing tissues and is regulated by the oxygen gradient [128]. HIF-1α is regulated by post-transcriptional control. The 3′ or 5′ UTR of HIF-1α mRNA contains AU-rich elements attracting the RNA binding protein, such as Human antigen R (HuR) or polypyrimidine tract-binding protein (PBP), for stabilizing HIF-1α mRNA and stimulating HIF-1α protein synthesis [129,130]. Similarly, the 3′ UTR of Trx mRNA is protected from degradation by the cold-inducible RNA-binding protein (CIRP) hnRNPA18, which enhances Trx expression [131]. Such post-transcriptional regulation is found in the mRNA of redox-embryogenesis related enzymes, including Cox-2, GPx1 and MnSOD (manganese-dependent superoxide dismutase or mitochondrial SOD2) [76].

### 5.4. Growth Factors and Redox Modulation 

Growth factors contribute to cell proliferation and differentiation in embryogenesis [132]. The signaling of the epidermal growth factor receptor (EGFR) shows a redox-regulated linkage to tyrosine kinase signaling, which is redox-sensitive [133]. Fibroblast growth factor (FGF) and vascular endothelial growth factor (VEGF) are inhibited in hyperoxia, leading to bronchopulmonary dysplasia in humans, demonstrating a linkage between ROS, growth factors and lung development [75]. H_2_O_2_-induced pulmonary hypertension in rats, which is modulated by EGFR signaling through redox regulation [134]. 

Vasculogenesis is an important event during early mammalian embryogenesis. In order to supply nutrients for embryos, blood vessels are stimulated for growing, branching and invading developing tissues and organs [135]. Crosstalk between growth factors and redox-related transcription factors promotes vascular development. HIF-1 responds to systemic oxygen changes, activates vascular endothelial growth factor (VEGF) transcription and modulates vascular development for adaption to hypoxia. HIF-1 is protective during embryogenesis, as demonstrated by preventing the lethality of mice at the embryonic 10.5 stage (E10.5) [56,136]. 

### 5.5. Redox Status and Signaling Pathways

The redox status plays a critical role in regulating differentiation, proliferation, apoptosis and cell division asymmetry during embryogenesis [2]. Therefore, redox signaling is required for building a healthy embryo at the correct time point and place. The evolutionally conserved regulation of the O_2_ concentration controls cell metabolic processes by producing, sensing and responding to its metabolites (ROS). This regulation contributes to redox cellular signaling. This network of redox cellular signaling not only involves cell metabolism but also embryogenesis [137]. ES cells undergo cell cycle arrest of the G2/M cell cycle phase transiently by increasing ROS levels, which indicates the oxidative stress resistance of the ESCs [138]. ES cells at an O_2_ concentration of 2–5% maintain their clonal regression and genetic integrity [139,140]. Lower O_2_ concentrations or oxidative stress induced by H_2_O_2_ leads to ES cell death by activating apoptotic signal transduction [138,140], indicating the importance of maintaining a physiological O_2_ concentration for regulating redox signaling for directing ES cells into the correct timeline of the cell cycle.

The Wingless and int-1 (Wnt) signaling pathway regulates embryonic cell differentiation, proliferation, cell fate determination and apoptosis, especially at the gastrulation stage [141,142]. Wnt signaling is activated by sensing the ROS level. Nucleoredoxin (Nrx) acts as a key regulator for Wnt signaling regulation by targeting dishevelled (Dvl) activity directly [105]. Hydrogen peroxide oxidizes Nrx, leading to Dvl release from the Nrx/Dvl complex, and promotes β-catenin accumulation to activate Wnt signaling [104]. Differential levels of Nrx regulate Wnt signaling during Xenopus gastrulation [103]. 

## 6. Conclusions

Maintaining redox homeostasis is critical for the normal development of an embryo. NADPH produced from the action of G6PD is important in maintaining redox homeostasis by serving as a cofactor for NADPH-dependent redox enzymes, including GSH reductase, TrxR, aldo-keto reductase, as well as NOX and NOS. GSH and TRX function as anti-oxidants that contribute to the maintenance of redox homeostasis, while ROS and RNS at physiologic concentrations (eustress) contribute to cellular growth and differentiation. G6PD is involved in oogenesis, embryogenesis and post-embryonic development. This is substantiated from studies in which G6PD is inactive or knocked-down. G6PD-deficient cells and organisms have been used as models for determining the role of redox signaling in regulating cell proliferation, differentiation and migration during embryogenesis. Embryogenesis is also modulated by anti-oxidant enzymes, transcription factors, microRNAs, growth factors and signaling pathways, which are dependent on redox regulation. Novel technologies, including multi-omics sequencing [143] and single-cell sequencing [144], are promising for comprehensively uncovering the molecular actions of how redox homeostasis regulates embryogenesis. Recent development of genetic engineering tools, such as CRISPR knock-in, can further establish new G6PD models in stem cells and model organisms [145,146,147]. It is anticipated that other redox-sensitive pathways governing embryogenesis will be reported following the development of novel and advanced technologies. 

## Figures and Tables

**Table 1:** Summary of redox-related factors and pathways in embryonic development.

Type	Name	Model	Functions	Experimental Protocols	References
Enzymatic/anti-oxidant enzyme	SODs	Human	The SOD activity in placenta protects embryos from lipid peroxidation.	Biochemical analysis	[77,78]
Enzymatic/anti-oxidant enzyme	MnSOD	Mouse *Drosophila*	Maintains cardiac function and neonatal survival. Prevents abnormal brain morphology and axonal aberrations during neurodevelopment.	Mouse: biochemical analysis. *Drosophila*: lifespan analysis, brain/neuron morphology	[73,79]
Enzymatic/anti-oxidant enzyme	GPx4	Mouse	Male reproduction and early embryonic development. Initiates gastrulation and develops embryonic cavities	siRNA knockdown. Molecular biology/morphology analysis.	[13,16,80]
Enzymatic/anti-oxidant enzyme	Prx	Human Cow *Drosophila* *C. elegans*	Stimulates blastocyst development and increases embryonic mitochondrial activity. Support embryogenesis.	Molecular biology/biochemical/life span analysis.	[31,81,82,83,84]
Enzymatic/anti-oxidant enzyme	Trx/TrxR	Mouse	Support embryonic viability and embryogenesis.	Knockout mice. Molecular biology/morphology analysis.	[10,11,12,13]
Enzymatic/pro-oxidant enzyme	NOX	Mouse	Promotes cardiac differentiation, cardiomyogenesis, and neonatal cardiac cell growth.	shRNA/siRNA knockdown. Biochemical/molecular biology/morphology analysis.	[66,85]
Non-enzymatic/transcription factor	Nrf2	Human Mouse	Enhances oxidative stress resistance. Controls self-renewal and pluripotency of ES cells. Supports embryo cleavage and blastocyst formation. Maintains stemness and survival under oxidative stress.	siRNA knockdown and CRISPR-mediated ectopic gene expression	[86,87,88,89]
Non-enzymatic/transcription factor	NF-κB	Mouse	Embryonic and liver parenchymal cell survival	Knockout mice coupled with histological/molecular biology analysis.	[90]
Non-enzymatic/transcription factor	AP-1	Mouse	Embryonic survival, liver erythropoiesis, hepatogenesis, development of the placenta and yolk sac, chondrocytes and extraembryonic tissues	Knockout mice coupled with biochemical/molecular biology/morphology analyses.	[91,92,93,94,95]
Non-enzymatic/transcription factor	HIF-1	Human mouse	Modulates vascular development (VEGF) in hypoxia. Trophoblast proliferation and differentiation, morphogenesis of the developing heart, chondrogenesis and myocardial development.	Knockout mice. Molecular biology/morphology analysis.	[96,97,98,99]
Non-enzymatic/transcription factor	*Pitx2, Pitx3*	Mouse	Anti-oxidant defense in fetal myogenesis	Knockout/knockdown and ectopic gene expression. Metabolomics/molecular biology analysis.	[100]
Non-enzymatic/post-transcriptional control	Pitx-miRNA (mir-15b, mir -23b, mir-106b and mir-503) pathway	Mouse	Modulates cell proliferation and cell fate of skeletal-muscle stem cells (satellite cells)	Knockout/knockdown and ectopic gene expression. Bioinformatics/molecular biology analysis.	[101]
Non-enzymatic/post-transcriptional control	Grsf-1	Mouse	Modulates brain development by recruiting *GPx4* mRNA to translationally active polysome fractions by targeting 5′ untranslated region (UTR) of *GPx4* mRNAs.	Bioinformatics/biochemical analysis	[102]
Signaling pathway	Wnt signaling (Nrx and Dvl)	Human *C. aethiops* Mouse *Xenopus*	Senses ROS levels to activate or inactivate Wnt signaling for directing embryo gastrulation.	siRNA knockdown and microinjection-mediated ectopic gene expression research. Biochemical/molecular biology/morphology analysis.	[103,104,105]
Signaling pathway	PPP pathway (including G6PD)	Mouse Zebrafish *C. elegans*	Maintains redox homeostasis during embryogenesis. Mediates abnormal lung development in hyperoxia in the neonatal mice.	Knockout/siRNA or morpholino knockdown. Metabolomics/biochemical/molecular biology/morphology analysis.	[23,34,35,36,37,38,39,40,41,42,43,44,46,47,48]
